# Endovascular treatment of haemorrhagic pancreatic serous cystadenoma

**DOI:** 10.1186/s42155-022-00343-w

**Published:** 2022-12-14

**Authors:** Thomas Le Tat, Robert Carlier, Mostafa El Hajjam, Guillaume-Marie Sarrot, Ilan Obadia, Mickael Tordjman, Jeffery Zhou

**Affiliations:** Ambroise Paré Hospital (AP-HP), Imaging Department, 9 Av. Charles de Gaulle, 92100 Boulogne-Billancourt, France

## Abstract

**Background:**

Pancreatic microcystic serous cystadenoma are rare benign pancreatic tumors. No treatment is needed in most cases as this lesion is often discovered incidentally. Surgery is not required except in symptomatic cases.

**Case presentation:**

We report herein a rare case of pancreatic serous cystadenoma complicated with a hemorrhage in a 95 years old patient treated with arterial embolization since surgery was not possible. The patient recovered without any adverse events or bleed recurrence in the 6 months following the procedure.

**Conclusion:**

Hemorrhage secondary to a pancreatic serous cystadenoma was successfully treated with arterial embolization, which may represent an alternative therapeutic option to surgery.

## Introduction

Pancreatic microcystic serous cystadenoma (SC) are rare benign pancreatic tumors, occurring mostly in middle age to elderly women. No treatment is needed in most cases as this lesion is often discovered incidentally (Jais et al. 2016). Surgery is usually not required except in cases of splenic vessels or common bile duct compression (European Study Group on Cystic Tumours of the Pancreas 2018). We report herein a rare case of a pancreatic SC complicated with massive hemoperitoneum.

## Case presentation

A 95-year-old woman was admitted to our emergency department for an episode of vomiting followed by loss of consciousness. During initial clinical examination, she presented with hypotension with a blood pressure of 70/40 mmHg and severe abdominal pain.

**Fig. 1:**
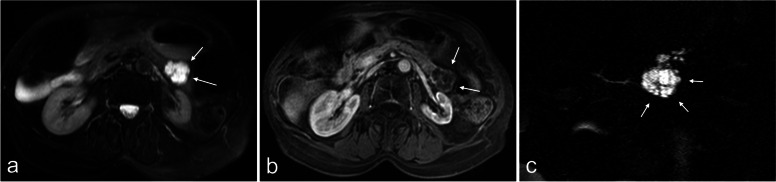
Pancreatic MRI performed 13 years earlier showed a typical pancreatic microcystic serous cystadenoma in the tail of pancreas. **a** Axial T2-weighted image. Well-defined, homogeneous, high T2 signal intensity, polycystic components (arrows). **b** Axial T1-weighted image after gadolinium chelates (Dotarem®) injection. Isolated septa enhancement (arrows). **c** Radial MR cholangiopancreatography. Honeycomb pattern (arrows)

**Fig. 2:**
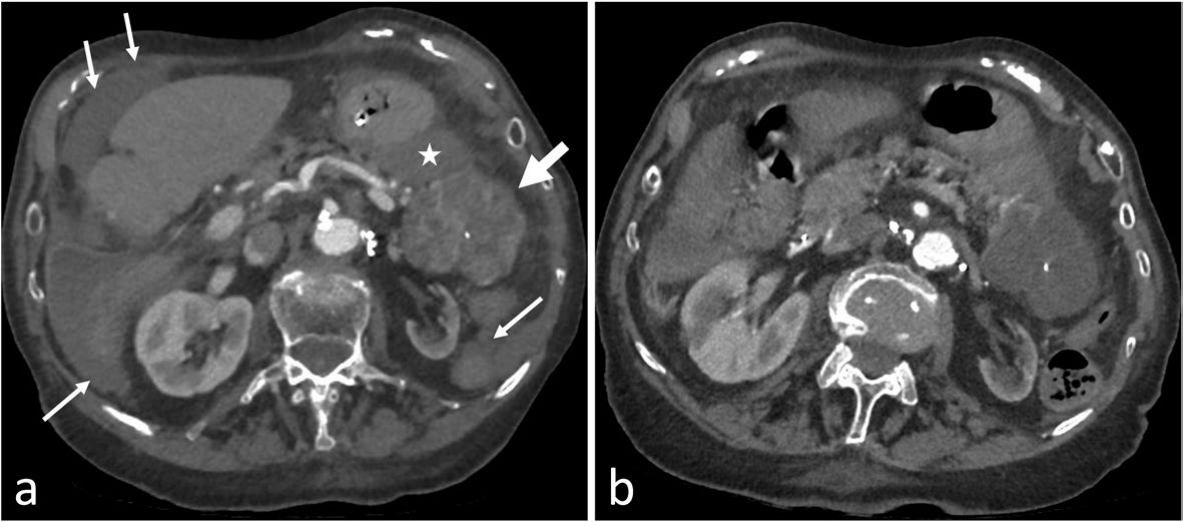
Axial CT-scan images, at the late arterial phase, before (**a**) and after (**b**) embolization procedure. **a** Initial CT-scan revealed a hematoma (star) next to the pancreatic tumor (thick arrow) and diffuse hemoperitoneum (thin arrows). Note the hypervascularisation of the tumor septa and the central calcification, both characteristic of serous cystadenoma. The lesion doubled in size in thirteen years (66 mm vs 31 mm). **b** CT-scan 2 days after embolization confirmed the disappearance of the hypervascularization of the tumor, with no signs of further bleeding

She had a medical history of pancreatic microcystic serous cystadenoma, stage III chronic renal failure secondary to renal artery stenosis and paroxysmal atrial fibrillation on Eliquis® (apixaban). A pancreatic MRI performed 13 years earlier showed a 3.1 cm mass in the tail of the pancreas with a microcystic high T2 signal intensity, with enhancing septa, that didn’t communicate with the main pancreatic duct or its branches, typical of pancreatic SC (Fig. 1), with no follow-up needed. The initial blood test revealed a hemoglobin level of 10.8 g/dL and a platelet count of 231,000/mL. Prothrombin time was slightly lowered, at 88%, and activated partial thromboplastin time ratio was normal at 0.76. Computed tomography (CT) (Definition AS+ 128, Siemens) revealed a large hemoperitoneum, an hematoma next to the previously known mass which had doubled in size in 13 years (6.6 cm), and enhancing septations with small peripheral contrast blush (Fig. 2a). No peritoneal arterial bleeding was visible on the CT. Due to the significant operative risks in this elderly patient, surgery was not proposed. However, to avoid further potential bleeding which could become quickly life-threatening in this very elderly patient, and to be able to restart the anticoagulant treatment, a hemostatic embolization of the tumor was decided and performed immediately.

**Fig. 3:**
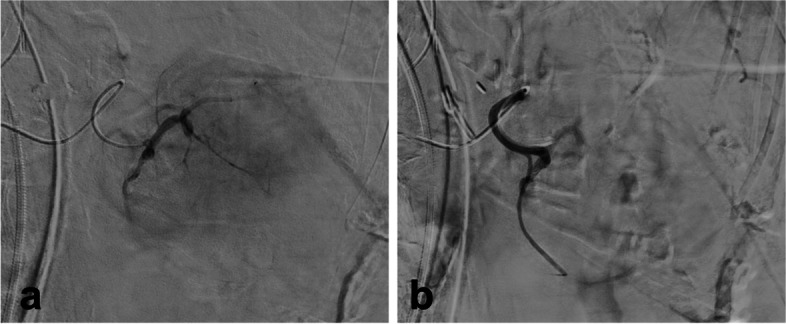
**a** Selective angiography of tumor-feeding vessel showed the hypervascular lesion with a tumor blush. **b** Angiography of the tumor-feeding vessel after embolization

Selective catheterization of the tumor-feeding vessel arising from the splenic artery was performed by a microcatheter (Progreat 2.4, Terumo) and a hydrophilic guidewire (GT45, Terumo) (Fig. 3), demonstrated the hypervascular nature of the lesion, and didn’t find any arterial bleeding. Embolization of the pancreatic tumor was performed using 500-700 μm microspheres (Embogold, Meritmedical). Microspheres were chosen due to the high vessel tortuosity, as the patient was very atherosclerotic, and had a surgical history of supra-celiac aorto-celiac graft with reimplantation of both renal arteries.

Endpoint of embolization was near stasis of blood flow in the abnormal vessels and disappearance of the tumor blush.

After embolization procedure, the hemoglobin level was stable at 8 g/dL. Follow-up CT-scan at 2 days (Fig. 2b) showed no signs of further bleeding. Anticoagulation could be resumed 48 hours after the procedure. No complications such as pancreatitis occurred during follow up, and 1 week after embolization, the patient was discharged home. No event or further bleeding occurred and follow-up CT-scan at 2 months showed no tumor growth. The patient presented no complication or recurrence at 6 months after the procedure.

## Discussion

Pancreatic SC are asymptomatic in most cases, becoming symptomatic in 11% of cases (Jais et al. 2016). Internal tumor bleeding is an unusual complication though, and hemoperitoneum is exceptional. Bleeding of these cystic lesions could be explained by the fibrous septa which are highly vascular when stained with the CD31 vascular marker, particularly in the microcystic SC. This could explain why some SC are hypervascular while being cystic lesions (Erkan 2019) In our case, anticoagulant treatment may have played a role in this hemorrhage.

Only three cases of hemoperitoneum secondary to pancreatic SC have been reported in the literature (Amaral et al. 2020; Ashkzaran et al. 2007; Cha et al. 2021). The first patient was treated by embolization followed by surgery (Amaral et al. 2020), the second one was treated by surgery alone (Ashkzaran et al. 2007), and both showed no malignancy on microscopic evaluation. The third one also had surgical resection, and synchronous pancreatic neoplasm was found out in pathology, so surgery should be recommended when possible (Cha et al. 2021).

Thus, in poor surgical candidates with hemorrhagic SC, such as our elderly patient, arterial embolization may be a safe and effective alternative to achieve hemostasis. Prominent arteries within the pancreatic tumor allowed for selective tumor embolization with a good clinical outcome without causing necrotizing pancreatitis, nor bleeding recurrence after few months.

## Conclusion

Hemorrhage secondary to a pancreatic serous cystadenoma was successfully treated with arterial embolization, which may represent an alternative therapeutic option to surgery.

## Data Availability

Not applicable.

## Abbreviations

SC: Serous cystadenoma
CT: Computed tomography
